# Low Oxygen Stress During Early Development Influences Regulation of Hypoxia-Response Genes in Farmed Atlantic Salmon (*Salmo salar*)

**DOI:** 10.1534/g3.120.401459

**Published:** 2020-07-07

**Authors:** Tara Kelly, Hanne Johnsen, Erik Burgerhout, Helge Tveiten, Tina Thesslund, Øivind Andersen, Nicholas Robinson

**Affiliations:** * Sustainable Aquaculture Laboratory- Temperate and Tropical (SALTT), School of BioSciences, The University of Melbourne, Parkville 3010, Australia; ^†^Nofima, N-9291 Tromsø, Norway; ^‡^Department of Animal and Aquacultural Sciences, Norwegian University of Life Sciences (NMBU), N-1433, Ås, Norway

**Keywords:** salmon, hypoxia, gene regulation, prolyl hydroxylase domain enzymes, DNA Methylation

## Abstract

Survival and growth of developing salmonids are negatively affected by low oxygen levels within gravel nests in natural streams, and hypoxic stress is often experienced by farmed Atlantic salmon (*Salmo salar*) within hatcheries. Exposure to hypoxia during early development may have long-lasting effects by altering epigenetic marks and gene expression in oxygen regulatory pathways. Here, we examine the transcriptomic response to low dissolved oxygen (DO) in post-hatch salmon reared continuously in 30%, 60% or 100% DO from fertilization until start of feeding. RNA sequencing revealed multiple differentially expressed genes, including oxygen transporting hemoglobin embryonic α subunit (*hbae*) and EGLN3 family hypoxia-inducible factor 3 (*egln3*) which regulates the stability of hypoxia inducible factor 1α (HIF-1α). Both *hbae* and *egln3* displayed expression levels inversely correlated to oxygen concentration, and DNA methylation patterns within the *egln3* promoter were negatively associated with the transcript levels. These results suggest that epigenetic processes are influenced by low oxygen levels during early development in Atlantic salmon to upregulate hypoxia-response genes.

Anadromous salmonids encounter a fluctuating physicochemical environment and have varying oxygen needs during the life cycle. High temperature and low oxygen levels may have detrimental effects on growth and survival of the developing embryo within gravel nests, and the aerobic capacity of adult fish may become restricted during challenging migration up- river from the sea to spawning grounds (Rubin and Glimsäter 1996; Youngson *et al.* 2004; Greig *et al.* 2007). Dissolved oxygen (DO) plays vital roles in maintaining biochemical and physiological processes, and hypoxic conditions may thereby limit energetic processes, and have detrimental effects on growth, reproduction and survival (Greig *et al.* 2006; Pedersen 1987; Randall *et al.* 1982; Rombough 1988; Richards 2009; Wang *et al.* 2009, 2016; Ho & Burggren 2012). The life cycle of Atlantic salmon (*Salmo salar*) is simulated in aquaculture by rearing the juvenile fish in freshwater tanks followed by the transfer of the post-smolt fish to sea-cages, where they are grown to harvest size. Hypoxic stress is often experienced by salmon, especially during the transfer to sea and within sea cages due to high stocking density or high water temperatures, and impaired health growth and survival has been reported in farmed salmon under hypoxic conditions (Hamor & Garside 1976; Remen *et al.* 2012, 2014; Kvamme *et al.* 2013; Wang *et al.* 2016; Vikeså *et al.* 2017; Iversen *et al.* 2005; Wood *et al.* 2019). There is a growing interest in whether hypoxic exposure at early life stages could influence the resilience of the fish to future hypoxic challenges.

Exposure to environmental stimuli, especially during early life stages, can prompt changes in epigenetic markers to shape the development and consequent phenotype of an organism (Best *et al.* 2018; Szyf 2013; Jaenisch & Bird 2003; Feil and Fraga 2012). When such changes increase the chance of survival, such a process is known as adaptive plasticity (Ghalambor, McKay, Carroll, & Reznick 2007). A growing focus in aquaculture research has been to limit the physiological effect of environmental stress, in order to improve fish health and production efficiency. This has led to an interest in the role of epigenetics in gene-environment interactions in aquaculture. Atlantic salmon exposed to environmental stress during early developmental stages show modified transcription and methylation associated with body growth and immune response (Moghadam *et al.* 2017; Uren-Webster *et al.* 2018). Parental hypoxic exposure in zebrafish (*Danio rerio*) increases hypoxia tolerance in offspring (Ho and Burggren 2012), rearing chinook salmon (*O. tshawytscha*) embryos in hypoxia improves fry tolerance to acute hypoxia stress (Del Rio, Davis, Fangue, & Todgham 2019) and the hatchery environment induces epigenetic modifications in coho salmon (*O. kisutch*) (Le Luyer *et al.*, 2017) and steelhead trout (*O. mykiss*) (Gavery *et al.*, 2019). Early exposure to chronic hypoxia in European sea bass (*Dicentrarchus labrax*) had short- and long-term effects on expression of hemoglobins, several hypoxia inducible factor 1 (HIF-1) related genes and egl-9 family hypoxia-inducible factor 3 *(egln3*), regulating the stability of the HIF-1α subunit (Vanderplancke *et al.* 2015; Cadiz *et al.* 2017).

In the current study, we investigated the epigenetic regulation of the hypoxic response in Atlantic salmon by analyzing the transcriptome and methylome in alevins after embryonic exposure to various chronic hypoxic treatments. This is part of a broader investigation of the potential to condition Atlantic salmon during early life stages for tolerance to hypoxia.

## Materials and Methods

### Experimental set up and sampling

Atlantic salmon eggs and milt were provided by AquaGen AS (Trondheim, Norway) and fertilized according to standard hatchery “dry fertilisation” protocols. Eggs from one female, including ovarian fluid, and milt from one male (2ml per liter egg) were gently mixed for 2 min prior to adding water. Milt and ovarian fluid were gently washed away, and eggs were left to swell and harden for 2 hr, then moved to incubators. Eggs that were unfertilized, underdeveloped, dead or ceased developing at the blastula stage, were visually identified and removed.

Immediately following fertilization, eggs were divided into three oxygen treatment groups *i)* 30% dissolved oxygen (30% O_2_) *ii)* 60% dissolved oxygen (60% O_2_) and *iii)* 100% dissolved oxygen (100% O_2_) and incubated in circular upwelling incubators (21cm diameter) at 7.3° ±0.3°, at 7.2° ±0.4° and at 6.7° ±0.3, respectively. Dissolved oxygen concentrations of 30% and 60% were within the range that salmon may encounter in the sea-cage environment (26–90%; Stehfest *et al.* 2017; Solstorm *et al.* 2018). The 100% dissolved oxygen condition was used as a control. The 30% dissolved oxygen concentration was achieved by degasification using pure nitrogen gas when trickling water through an approximately 1m^3^ box filled with plastic media to increase the surface area. The 60% saturation treatment was achieved by mixing untreated water and 30% saturated water. The process of degasification caused the slightly higher temperature for lower oxygen treatments as detailed above. Water flow was checked weekly and adjusted to approximately the same rate in all incubators (variance ± 0.1 L/min).

**Figure 1:**
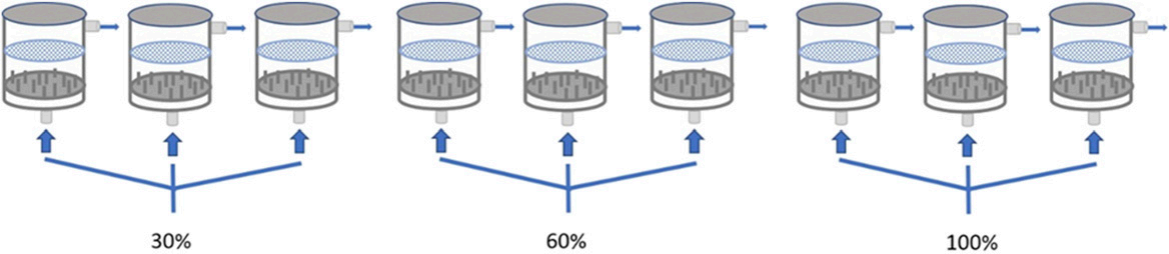
Schematic of upwelling incubator setup showing water flow (blue arrows) and oxygen saturation. Eggs rested upon perforated plates (blue). Once hatched, larvae swam through the openings and lay on a plastic substrate (gray). After hatching the perforated plate was removed.

Each treatment group was kept in triplicate tanks with approximately 1300 eggs per replicate. Eggs rested upon perforated plates (4mm diameter holes) within the incubators. Once hatched, larvae swam through the openings and rested on the plastic substrate (Figure 1).

At several timepoints throughout the alevin stage (Figure S1), individuals were randomly sampled and directly euthanised using an overdose of Benzocain (Benzoak Vet.). Whole alevin samples without yolk sac were placed in RNA-later (Ambion) and kept at 4° overnight before storage at -20°. A Kruskal-Wallis test was conducted in R (R Core Team 2019) to assess if weight differences between treatment groups was significant (n = 120, *P* < 0.05). Ethical guidelines supplied by the Norwegian Food Safety authority were followed. The guidelines state that experiments using fish prior to start-feeding are not subject to ethics approval.

### RNA and DNA extraction

Total RNA and DNA was isolated and purified from whole alevins without yolk sac using AllPrep DNA/RNA/miRNA Universal Kit (Qiagen) according to the manufacturer’s protocol. RNA quantity and quality were determined using nanodrop (Thermo Scientific) and bioanalyzer (Agilent) instruments. Isolated RNA with a 260/280 ratio >1.9 and RNA integrity number (RIN) >8, and DNA with a 260/280 ratio >1.8 was considered to be pure. Isolated DNA and RNA was stored until further analysis at -20° and -80°, respectively.

### RNA sequencing, alignment and differential expression analysis

At 925 degree days (DD), just before start of feeding, twelve alevins (4 per replicate) from each of the three oxygen treatment groups were used for RNA sequencing. We focused on this point of development because larvae up to this point had been solely dependent on their endogenous nutrient supply (yolk), and we were concerned that after the salmon lose the yolk sac gene expression patterns might be influenced by exogenous feeding. Due to the impact of low oxygen saturation on development, individuals reared in 30% O_2_ were visibly less developed at 925DD than those at 60% O_2_ and 100% O_2_, marked by less absorption of the yolk sac. To minimize developmental stage effects, an additional 12 individuals were randomly sampled from the 30% O_2_ treatment group at 990DD, which matched the same developmental stage (end of yolk sac absorption) as the other groups.

Illumina sequencing of the 48 RNA samples was conducted by the Norwegian Sequencing Centre (Oslo, Norway). Libraries were prepared using a Strand-specific TruSeq RNA-seq library preparation kit (Illumina), and paired-ends sequenced using a HiSeq 3/4000 Genome Analyzer (Illumina), in 4 lanes with a read length of 75bp.

Leading and trailing low quality or N bases (quality score <10) were removed from the sequence using Trimmomatic (Bolger *et al.* 2014). The reads were scanned using a 4-base window, and the window was cut when the average quality per base fell below 15. Reads less than 36 bases in length were removed from the analysis.

Reads were mapped to the Atlantic salmon genome assembly (GCA000233375.4 ICSASG_v2.) (Lien *et al.* 2016) using TopHat v.2.0.8b with default parameters other than b2 option – sensitive (Trapnell *et al.* 2009; Langmead *et al.* 2009). SamTools (Li *et al.* 2009) was used to sort the alignments and HtSeq-count was used to count how many reads mapped to each interval on each chromosome, skipping reads with alignment quality below 10 (Anders *et al.* 2015).

The DESeq2 package in R was used to quantify differential gene expression between oxygen treatments using a negative binomial linear model with default parameters (Love *et al.* 2014). Genes from the DESeq2 analysis with adjusted *p*-values < 0.05 were considered as differentially expressed. A complete linkage method was used to perform a hierarchical cluster analysis using the heatmap.2 and hclust packages in R.

### Enrichment analysis of Gene Ontology terms

The R package topGO (Alexa *et al.* 2006) was used to perform an enrichment analysis for Gene Ontology (GO) terms (Ashburner *et al.* 2000; The Gene Ontology Consortium 2019) using the list of differentially expressed genes detected between oxygen treatment groups against all genes in the annotated reference genome. Significance level was established using Fisher’s exact test (*P* < 0.05). GO annotations for *Salmo salar* reference sequence ICSASG_v2 were downloaded from GitHub (https://rdrr.io/github/FabianGrammes/Ssa.RefSeq.db/).

### qPCR gene expression analysis

Quantitative real-time PCR (qPCR) was used to investigate relative expression of important genes identified by RNA-seq at two additional sampling timepoints: 1) the point of hatching which occurred at around 560DD (and is therefore denoted here as 560DD) and 2) 700DD.

Total RNA was extracted from twelve individuals sampled per treatment group and cDNA was synthesized using the High-Capacity RNA-to-cDNA Kit (Applied Biosystems) according to manufacturer’s protocol, using 200ng of total RNA. Significantly differentially expressed genes with putative functions affecting hypoxic response were targeted for qPCR. Target genes were *egln3*, *egln2*, *egln1_L* and hemoglobin embryonic α (*hbae*). *Egln1_L* and *egln2* were not among the differentially expressed genes according to RNAseq analysis but were included as qPCR targets for comparison. Primers were designed using Primer Express 3 software (Life Technologies) (Table S1). To ensure the specificity of primers, paralogous gene sequences were aligned using MUSCLE (https://www.ebi.ac.uk/Tools/msa/muscle/), viewed using GenDoc Multiple Sequence Alignment Editor & Shading Utility Version 2.7 (https://genedoc.software.informer.com/2.7/), primers designed in areas of sequence difference between paralogs and primer sequences checked for secondary matches using BLAST at NCBI. Once the primers were ordered, checks were made for primer dimers, secondary amplification products and secondary melting curve peaks using standard methods.

A twofold standard curve of cDNA was prepared, combing 12 samples across all treatments and creating an eight-point dilution series. This was done for each primer set to determine amplification efficiency and a melting curve analysis was used to check for primer-dimers and untargeted amplification products. No template controls, and a control to confirm the absence of genomic DNA, were run. The qPCR was run in duplicates using QuantStudio 5 (Thermo Fisher). Each well contained Power SYBR Green (Thermo Fisher), 300nM final concentration of each primer, 7µL of diluted cDNA and nuclease free water (Ambion) to a final reaction volume of 20 µL.

Quant Studio Design & Analysis Software (Thermo Fisher), was used to collect and analyze all data. For each gene, the sample with the lowest expression (highest mean CT value) across all time points was used as what is denoted as a calibrator by (Pfaffl *et al.* 2004). Reference genes were used as internal controls to normalize expression data and remove non-biological variation. The reference genes *ef1α*, *18S* and *β-actin* were verified using BestKeeper software (Pfaffl *et al.* 2004) and the geometric mean of reference gene CT values was used to calculate relative expression of target genes (Vandesompele *et al.* 2002). Significance was tested using a Kruskal-Wallis test.

### Analysis of DNA methylation in egln3 promoter

Twelve samples from each oxygen treatment group at the 700DD sampling timepoint were used for analysis of DNA methylation percentage on a PyroMark Q24 advanced system (Qiagen) (n = 36). This sampling time point was chosen because we hypothesized that most of the effects of treatment on DNA methylation would have occurred by this stage and that levels of DNA methylation would persist until differential gene expression was assessed.

Bisulfite conversion of DNA was completed using a Epitect Fast Bisulfite Conversion Kit (Qiagen) according to the protocol. Two pyrosequencing assays were designed using a PyroMark Assay Design 2.0 (Qiagen) to cover five of the eight CpG sites in the Atlantic salmon *egln3* putative promoter region (Table S2). Primers could not be adequately designed to cover CpG sites 1-3. PCR was carried out using the PyroMark PCR Kit (Qiagen) according to the protocol with Mg^2+^ added to the reaction to improve the signal. Singular PCR products were confirmed on a 1% agarose gel. PyroMark Q24 Advanced CpG Reagents (Qiagen) were used along with Streptavidin Sepharose High Performance Beads (GE Healthcare). The percentage of methylation per CpG site was obtained by pyrosequencing performed on a PyroMark Q24 pyrosequencer following manufacturer’s protocol. Briefly, samples were first washed on a PyroMark Q24 Vacuum Workstation (Qiagen) using 70% ethanol, denatured using PyroMark Denaturation Solution (Qiagen) and washed in PyroMark Washing Buffer (Qiagen). Samples were then annealed with the sequencing primer in a PyroMark Q24 Plate (Qiagen), which was thereafter warmed for 5 min at 80° using a pre-heated PyroMark Q24 plate holder. Within 30 s, the heated plate containing single stranded template DNA with sequencing primer was transferred into the PyroMark Q24 Instrument and the run was started to collect the methylation data. Finally, PyroMark Q24 Advanced software was used to visualize and collect the methylation percentage per site and assess the quality of the data following manufacturer’s guidelines.

The Kruskal-Wallis test was used in R to determine if any of the five CpG sites in the *egln3* promoter showed significantly different methylation between the treatment groups (n = 12 per treatment, *P <* 0.05). Association between DNA methylation changes in the *egln3* promoter and relative abundance of *egln3* transcripts was examined using DNA and RNA extracted from the same individuals.

Methylation and expression data for the 36 individuals was collected and the *cor.test* package in R was used to assess correlation between the data, based on Pearson’s correlation coefficient, following a t-distribution with n-2 degrees of freedom and a significance threshold of 0.05.

### Data availability

RNA sequence data for this study has been submitted to the NCBI Sequence Read Archive (http://www.ncbi.nlm.nih.gov/sra under accession number PRJNA609825). File S1 contains results of differential gene expression analysis. File S2 contains all other supplementary figures and tables. Supplemental material available at figshare: https://doi.org/10.25387/g3.12587207.

## Results

### Alevin weight

**Figure 2:**
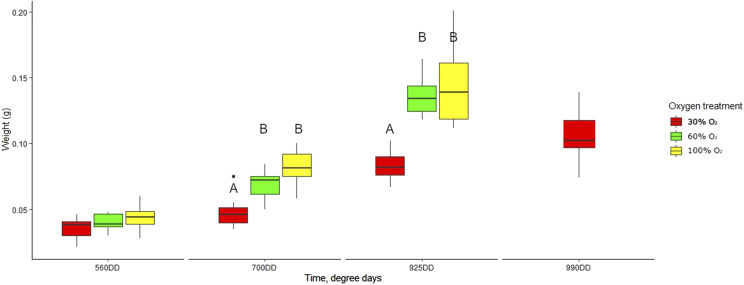
Weight (g) of Atlantic salmon alevins (without yolk sac) sampled for the various oxygen treatments and timepoints throughout the experiment. The 990DD stage of the 30% O_2_ group was included in the analyses due to slowed development as explained in M&M. Outliers are plotted as individual points. Letters indicate significant weight differences between treatment groups at each point of time (*P* < 0.05, Kruskal-Wallis test).

**Figure 3:**
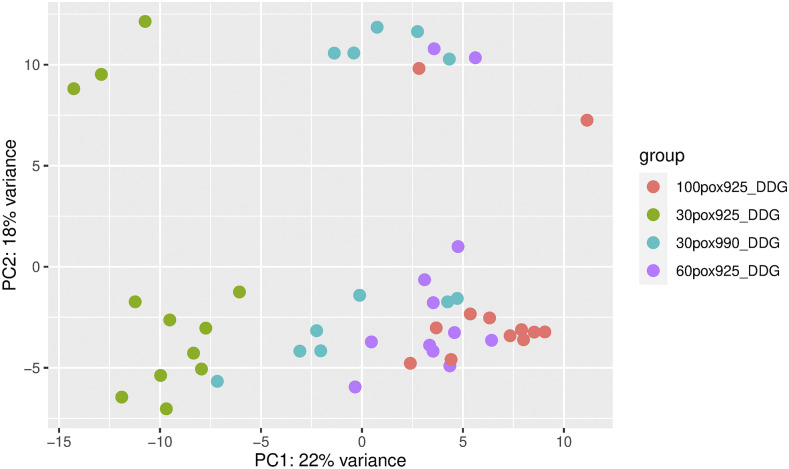
Principal component analysis of treatment at the stages of development sampled.

The weight of alevins was positively associated with dissolved oxygen across all timepoints (560DD, 700DD and 925DD, Figure 2). The weight disparity between the 30% O_2_ group and the other groups at 925DD (*P* < 0.05), in addition to the presence of a yolk sac in only the 30% O_2_ group at 925DD, indicate that this group was relatively underdeveloped at this timepoint. This was supported by the Principal Component Analysis (PCA) of the transcriptomes separating the 925DD 30% O_2_ cluster from the 925DD 60% O_2_, 925DD 100% and 990 30% O_2_ clusters (Figure 3, principal component 1). To reduce any potentially confounding effects of developmental stage we have focused mainly on the comparison of 30% O_2_ 990DD, 60% O_2_ 925DD and 100% O_2_ 925DD treated fish.

### Differential gene expression associated with oxygen treatment

**Figure 4:**
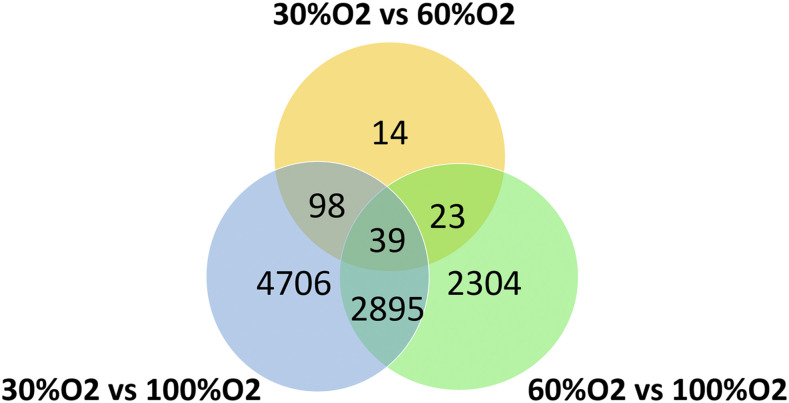
Representation of the abundance of differentially expressed genes between oxygen treatments at 925DD (990DD for 30% oxygen treatment group).

**Figure 5:**
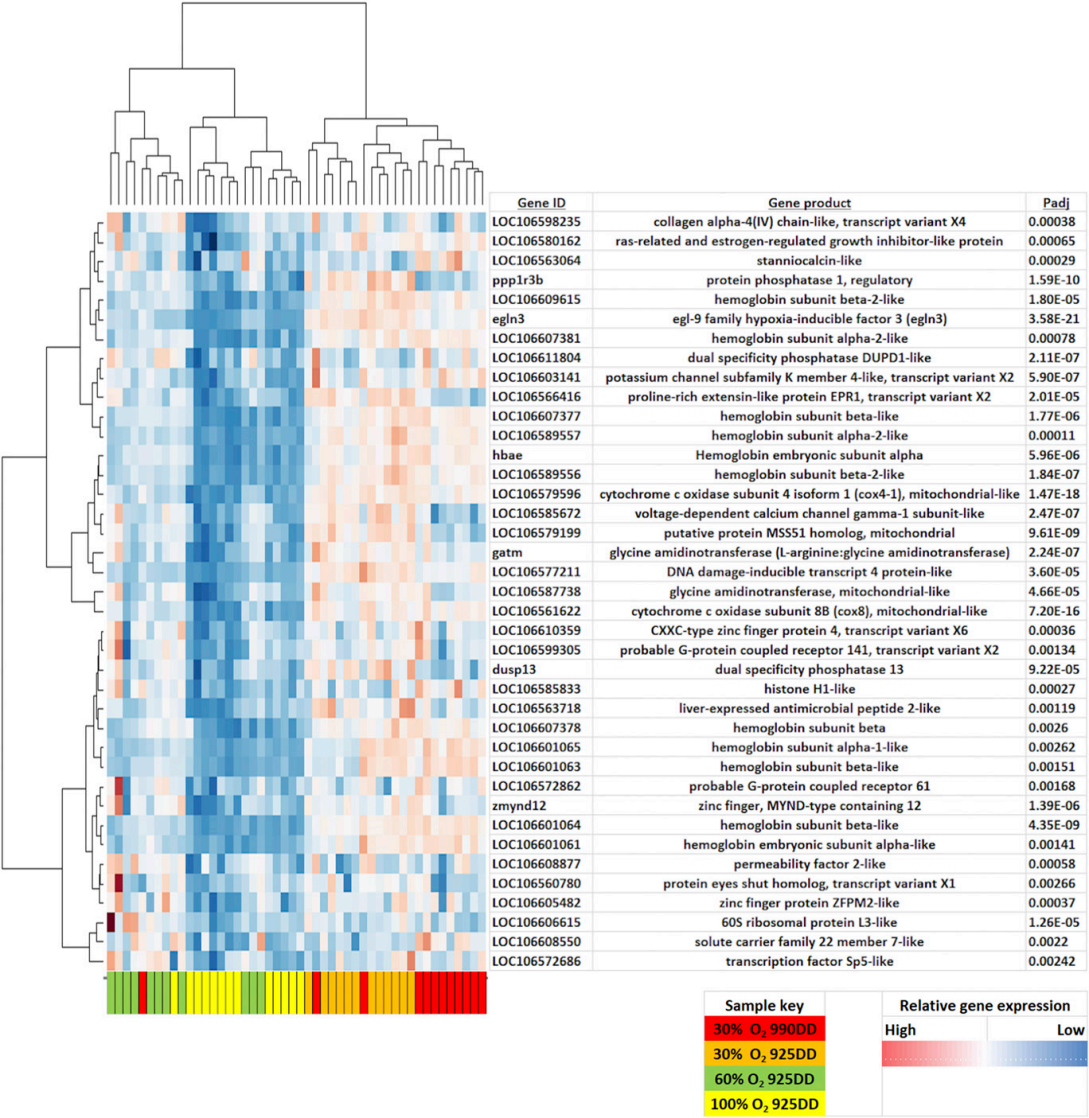
Heat map of differentially expressed genes (P_adj_ <0.05, fold-change >1.75) across salmon raised in 30% O_2_, 60% O_2_ or 100% O_2_ treatments.

Samples clustered according to oxygen treatment in the PCA with 30% O_2_, 60% O_2_ and 100% O_2_ treated fish distributed from left to right along the *x*-axis of the PCA plot (Figure 3). In this way PC1, which explained most (22%) of the variance, was positively associated with treatment oxygen level. The factor influencing PC2 which explained 18% of the variance was unknown. Although the unknown influencing factor on PC2 might reduce the power to detect differential expression associated with treatment, PC2 is clearly not confounded with treatment, and therefore should not be causing false positive results in the analysis of treatment effects. Analysis of RNAseq data (File S1) identified 39 genes that were systematically differentially expressed when comparing expression between the 900DD-30% O_2_ and 925DD-60% O_2_ treatments, 900DD-30% O_2_ and 925DD-100% O_2_ treatments and the 60% O_2_ and 100% O_2_ treatments (Figure 4). The expression of most of the 39 genes, including *egln3*, *hbae*, cytochrome c oxidase-like *cox4* and *cox8*, and DNA damage-inducible transcript 4-like (*ddit4*), were upregulated with reduced % O_2_ treatments in a dose-dependent manner (Figure 5, Table S3). Comparison of the two most extreme treatments (30% O_2_ *vs.* 100% O_2_) identified the largest numbers of differentially expressed genes (7738) and many of these genes (3032) were also differentially expressed in the other treatment comparisons. A lower number of differentially expressed genes (174) were detected for the 30% O_2_ to 60% O_2_ treatment comparison than for the other comparisons.

Gene Ontology terms that were significantly over-represented when comparing 60% O_2_ and 100% O_2_ treatments included “structural constituent of ribosome”, “oxidoreductase activity, acting on heme group of donors”, “heme-copper terminal oxidase activity” and “cytochrome-c oxidase activity”, including various cytochrome c oxidase subunit genes in the respiratory electron transport chain. Many of these terms contain genes typically involved in redox reactions (Table S4). The GO-terms were slightly different with comparison of 30% O_2_ to 60% O_2_ treatments, and additional terms such as “iron ion binding”, “cofactor binding”, “molecular carrier activity”, “oxygen binding” and “heme binding” were over-represented with this comparison (Table S4). A mixture of the same terms described for the two comparisons above were over-represented when comparing 30% O_2_ and 100% O_2_ treatments. Many new terms not observed in the above three comparisons, such as “catalytic activity” (including 2-oxoisovalerate dehydrogenase subunits), “isomerase activity” (including disulphide isomerases and enolase), “cofactor binding” and “translation factor activity” were over-represented when comparing 990DD and 925DD developmental stages for the 30% O_2_ treatment (Table S4). Some terms in common with the O_2_ treatment comparisons above were also over-represented in the comparison of 30% O_2_ treatment at 990DD and 925DD, such as “oxidoreductase activity”, “coenzyme binding” and “vitamin binding”. These terms therefore represent genes that responded differently to the 30% O_2_ treatment depending on developmental stage.

**Figure 6:**
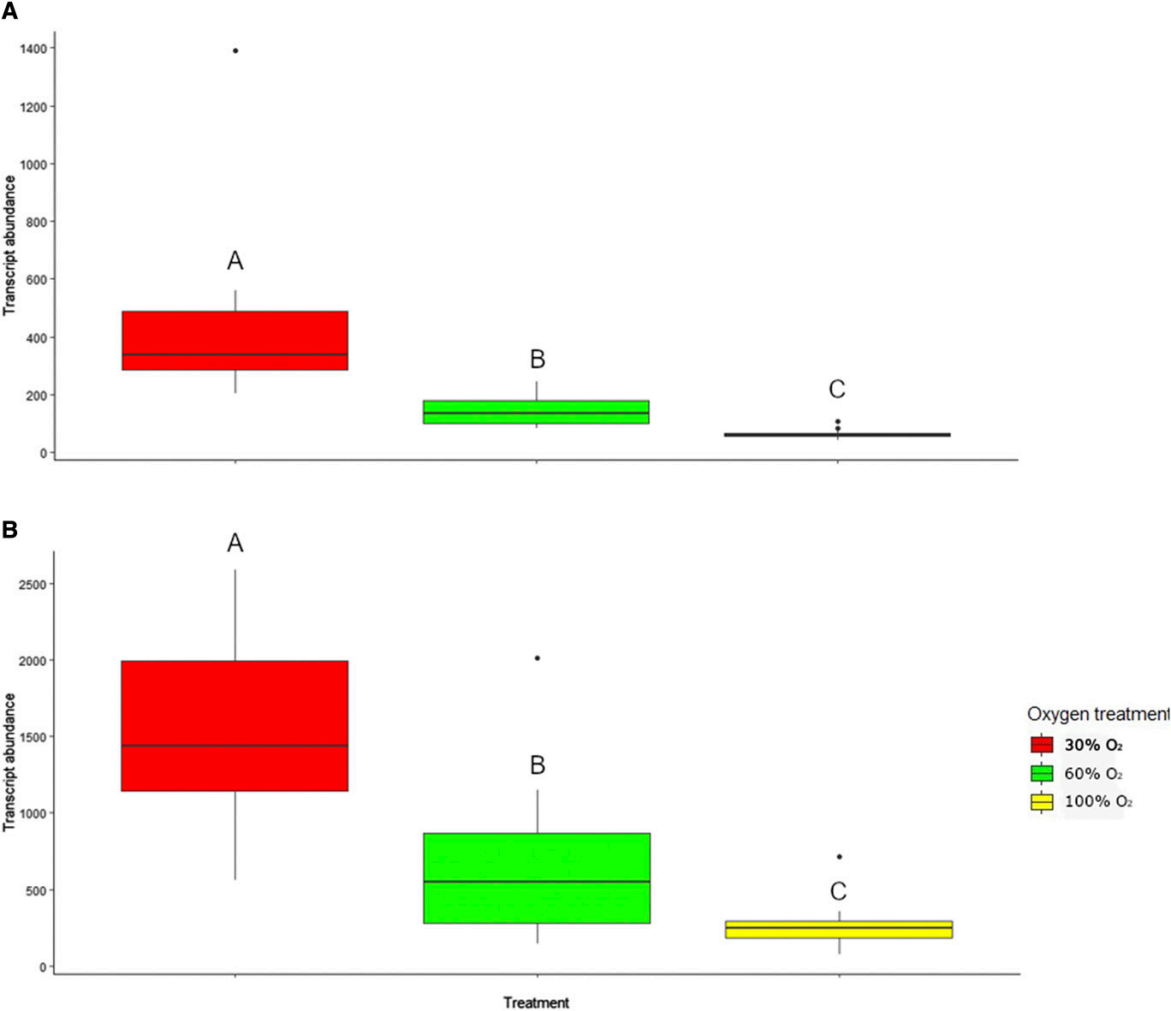
Transcript abundance (normalized counts, scaled to account for sequencing depth and RNA composition according to the method of Anders and Huber 2010) for A. *egln3* and B. *hbae* in Atlantic salmon reared in 30% O_2_, 60% O_2_ or 100% O_2_ at 925DD (990DD for 30% O_2_ group). Outliers are plotted as individual points. Letters indicate significant differential gene expression between treatment groups (*P* < 0.05).

We decided to focus on the 39 genes that were differentially expressed between all the O_2_ treatment comparisons, in particular the hypoxia-related genes *egln3* and *hbae* (Figure 5). *Egln3* (XM_014194431.1) had the most significant difference (lowest p-value) in transcript abundance between the three groups (with the highest transcript abundance in the 30% O_2_ group, followed by 60% O_2_, and then 100% O_2_ groups (Figure 6a). *Hbae* transcript levels (NM_001140924.1) showed a similar trend to that of *egln3* (Figure 6b). These trends also held at the earlier developmental stage of 30% O_2_ 925DD, but the e*gln3* transcript levels were slightly higher in 30% O_2_ 925DD individuals compared to 30% O_2_ 990DD individuals (non-significant, Figure S2).

**Figure 7:**
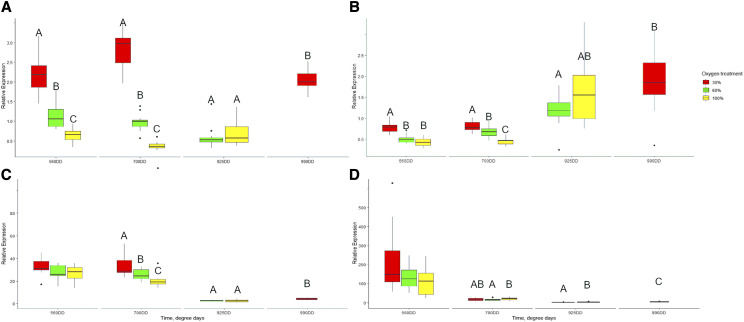
Relative gene expression (qPCR) of three Atlantic salmon *egln paralogs* and *hbae* in alevins at 560DD and 700DD. *Egln3* (a), *egln2* (b), *egln1L* (c) and *hbae* (d). Outliers are plotted as individual points. Letters indicate significant differential gene expression between treatment groups at each point of time (*P* < 0.05, Kruskal-Wallis test). Treatments for time points 925DD (60% and 100% O_2_) and 990DD (30% O_2_) were also compared.

The relative expression levels of *egln3*, *egln2*, *egln1_L* and *hbae* were quantified by qPCR in whole alevins at earlier stages 560DD (hatching) and 700DD. *Egln3* expression was negatively associated with the O_2_ levels at 560 and 700DD (Figure 7a). *Egln3* expression was significantly upregulated with the 30% DO treatment at 990DD compared to 60 and 100% treatments at 925DD (Figure 7a).

The relative expression levels of *egln2* and *egln1_L* followed a similar pattern to *egln3* at 560DD and 700DD (Figure 7b and c). *hbae* expression was reduced as development proceeded and showed significant upregulation with 30% DO treatment at 700DD and 990DD (Figure 7d).

### DNA methylation in egln3 promoter

**Figure 8:**
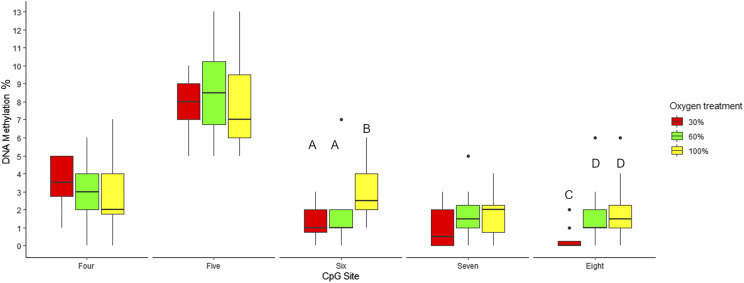
DNA methylation of CpG sites 4-8 in the *egln3* promotor region. DNA methylation (%) was measured at 700DD in fish raised under 30% O_2_, 60% O_2_ or 100% O_2_ treatments. Outliers are plotted as individual points. Letters indicate significant DNA methylation between treatment groups per site (*P* < 0.05).

The impact of low oxygen treatments on the DNA methylation of the CpG sites numbered 4-8 in the *egln3* promoter were investigated at 700DD. Methylation decreased at site 4 as oxygen treatment increased, while at site 5 the greatest average methylation was observed in the 60% O_2_ treatment group, with the 30% O_2_ and 100% O_2_ groups having a lower, but similar average methylation. A difference in methylation between treatment groups was detected at sites 6 and 8 (*P* < 0.05, Table S3, Figure 8).

**Figure 9:**
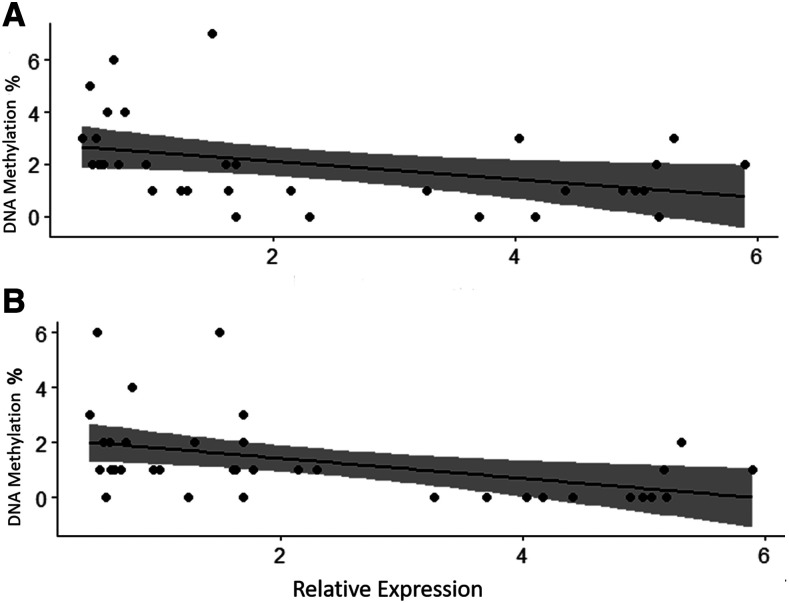
*Egln3* expression compared to DNA methylation in the promoter. Relative *egln3* RNA expression in salmon raised in 30% O_2_, 60% O_2_ or 100% O_2_ against DNA methylation (%) at the CpG sites 6 (A) and 8 (B) of the *egln3* gene promoter in alevins at 700DD (n = 36).

The correlation between *egln3* expression and DNA methylation at promoter CpG site 6 (*r* = -0.497, *P* < 0.01) and CpG site 8 (*r* = -0.494, *P* < 0.01) was negative and moderate in strength (Figure 9). In addition, the correlation between expression and methylation at CpG sites 6 and 8 combined was significant (*r* = -0.56, *P* < 0.01) and greater than that at sites 6 and 8 alone.

## Discussion

Environmental stresses such as low dissolved oxygen during early life can influence phenotype and can potentially affect the animal’s response to this stressor at later stages (Luo and Wang 2018; Nanduri *et al.* 2017; Hancock *et al.* 2015). Our results show that low oxygen treatments during early development of farmed Atlantic salmon have a significant effect on transcript expression of hypoxic-response genes. The negative correlation between *egln3* expression and DNA methylation in two CpG sites of the *egln3* promoter suggests that hypoxic treatments trigger changes in DNA methylation within the promoter to upregulate *egln3*. Accordingly, CpG methylation of the human *egln3* homolog was found to prevent hypoxia inducible factor (HIF)-1α degradation in plasma cell neoplasia leading to the activation of HIF target genes (Shah *et al.* 2007; Hatzimichael *et al.* 2010). Acute low oxygen treatments (1% O_2_ for 1-2 h) have been found to induce *egln3* expression during the first 24 h of development in zebrafish (Manchenkov *et al.* 2015) at a stage when the HIF pathway has been considered inactive (Mendelsohn and Gitlin 2008; Robertson *et al.* 2014). Applying extreme acute hypoxic treatments (0.5% and 5% dissolved oxygen for 4 h) to zebrafish embryos induced the HIF-1 oxygen response pathway and improved hypoxia tolerance in larvae, however this improved tolerance was not observed to persist in juvenile and adult developmental stages (Robertson *et al.* 2014). Together these studies indicate that the upregulation of *egln* family genes induced during treatment of the embryo may persist after the alevin stage of development and suggest that CpG methylation of the *egln3* promoter contributes to regulate the expression level of the gene. Methylation analysis of juvenile and adult salmon will help to assess the potential for longer-lasting gene expression and methylation changes and determine whether such treatments could condition the salmon epigenome for resilience to hypoxia.

The EGLN enzymes, or mammalian PHD homologs, regulate the stability of HIF during normoxic conditions by targeting HIF-1α for degradation (Appelhoff *et al.* 2004; Maxwell *et al.* 1999; Ivan *et al.* 2001; Jaakkola *et al.* 2001; Smith *et al.* 2008). The function of ELGN enzymes is inhibited in the absence of oxygen, and the consequent formation of the HIF complex promotes the transcription of several hundred hypoxia-response genes in order to reduce the effects of hypoxia. The expression of *egln3*, *hbae*, *egln2*, *egln1_L*, *cox* (subunit 4 isoform 1 and subunit 8) and *ddit4* was consistently highest in salmon alevins raised under severe hypoxic conditions (30% O_2_). Similarly, the significant hypoxia-induced expression of three hemoglobin genes in European sea bass was associated with stimulation of *egln3* (Cadiz *et al.* 2017). Work with alveolar type II epithelial cell cultures showed that the regulation of hemoglobin gene expression is likely to be indirectly regulated by upstream transcriptional activation by the HIF complex (Grek *et al.* 2011). Salmonids possess multiple hemoglobin isoforms with distinct functional properties and several isoforms are produced at specific developmental stages (Andersen 2020). Embryonic hemoglobins are involved in oxygen transport during early developmental stages (Maruyama *et al.* 2002), and salmon *hbae* was one of eleven hemoglobin subunit transcripts that were differentially expressed across the three O_2_ treatments. Moreover, differential regulation of hemoglobin isoforms in response to hypoxia has been observed in various other fish species, such as red drum (*Sciaenops ocellatus*) and the Lake Victoria cichlid *Haplochromis ishmaeli*. This so-called ’hemoglobin isoform switching’ is associated with increasing Hb- O_2_ affinity and may safeguard oxygen transport in fish during hypoxic events (Rutjes *et al.* 2007; Pan *et al.* 2017).

Hypoxic regulation of the HIF axis has been shown to influence gene regulation and physiology of several other downstream biological pathways. Here we show that cytochrome c oxidase *cox4-1* and *cox8* together with *ddit4* were significantly up-regulated in expression as oxygen treatment concentrations decreased (File S1). DDIT4 promotes survival under chronic stress conditions such as hypoxia, and *ddit4* expression is induced by HIF-3 in human endothelial cells during prolonged severe hypoxia (Hay and Sonenberg 2004; Janaszak-Jasiecka *et al.* 2016). Episodic hypoxic treatment of European sea bass larvae was associated with altered metabolism in juvenile stage fish (Vanderplancke *et al.* 2015) and chronic exposure of zebrafish parents to hypoxia for several weeks led to significantly increased hypoxic resistance and body length in larval offspring (Ho & Burggren 2012). Hypoxic treatments of rainbow trout embryo also affect subsequent growth and glucose metabolism (Liu *et al.* 2017a, b). Exposure of Atlantic salmon embryos to hypoxia prior to hatching resulted in improved hypoxia tolerance of the hatchling, while hypoxia treatments during alevin development did not affect hypoxia tolerance of the alevin (Wood *et al.* 2019). Hence, the effects of hypoxia seem to depend on the frequency and duration and what developmental the treatment is applied and when the effects are measured. Further studies are needed to assess the hypoxic tolerance of treated fish at later life stages in response to sustained low oxygen treatment, and whether this might condition the fish for improved tolerance to low oxygen environments.

## Conclusion

Our study has demonstrated that the use of hypoxia as a treatment during the early life stages of Atlantic salmon has a significant effect on DNA methylation and upregulation of the expression of hypoxia-response genes that play a key role in HIF-mediated oxygen homeostasis. These results, along with other salmonid studies reporting increased tolerance in hatched larvae after embryonic hypoxia treatment, demonstrates the potential for environmental stimuli to be utilized as treatments to shape gene regulation in farmed Atlantic salmon.

As hypoxia in the farming environment of Atlantic salmon is a key contributor to mortality (Randall *et al.* 1982; Iversen *et al.* 2005) continuing research to improve hypoxic tolerance is necessary. This and other studies have verified that low oxygen treatments can trigger an epigenetic response that upregulates genes in the HIF-1 hypoxia response pathway. Whether or not this has the potential to improve tolerance to hypoxia, and the effects of such treatments on the response of the fish to other environmental stressors (*e.g.*, immune response to disease), general survival and other side-effects will need to be investigated more thoroughly.

## References

[bib1] Alexa A., Rahnenfuhrer J., and Lengauer T., 2006 Improved scoring of functional groups from gene expression data by decorrelating GO graph structure. Bioinformatics 22: 1600–1607. 10.1093/bioinformatics/btl140 16606683

[bib2] Anders S., and Huber W., 2010 Differential expression analysis for sequence count data. Genome Biol. 11: R106 10.1186/gb-2010-11-10-r106 20979621 PMC3218662

[bib3] Anders S., Pyl P. T., and Huber W., 2015 HTSeq- A Python framework to work with high-throughput sequencing data. Bioinformatics 31: 166–169. 10.1093/bioinformatics/btu638 25260700 PMC4287950

[bib4] Andersen Ø., 2020 Multiplicity and polymorphism of fish hemoglobins, pp. 323–344 in *Vertebrate and Invertebrate Respiratory Proteins*, *Lipoproteins and Other Body Fluid Proteins* (Subcellular Biochemistry, Vol. 94), edited by Hoeger U. and Harris R. J. Springer International Publishing, Cham, Switzerland. 10.1007/978-3-030-41769-7_13 32189306

[bib5] Appelhoff R. J., Tian Y., Raval R. R., Turley H., Harris A. L., 2004 Differential Function of the Prolyl Hydroxylases PHD1, PHD2 and PHD3 in the Regulation of Hypoxia-inducible Factor. J. Biol. Chem. 279: 38458–38465. 10.1074/jbc.M406026200 15247232

[bib6] Ashburner M., Ball C. A., Blake J. A., Botstein D., Butler H., 2000 Gene ontology: tool for the unification of biology. Nat. Genet. 25: 25–29. 10.1038/75556 10802651 PMC3037419

[bib7] Best C., Ikert H., Kostyniuk D. J., Craig P. M., Navarro-Martin L., 2018 Epigenetics in teleost fish: From molecular mechanisms to physiological phenotypes. Comp. Biochem. Physiol. B Biochem. Mol. Biol. 224: 210–244. 10.1016/j.cbpb.2018.01.006 29369794

[bib9] Bolger A. M., Lohse M., and Usadel B., 2014 Trimmomatic: a flexible trimmer for Illumina sequence data. Bioinformatics 30: 2114–2120. 10.1093/bioinformatics/btu170 24695404 PMC4103590

[bib10] Cadiz L., Servili A., Quazuguel P., Madec L., Zambonino-Infante J., 2017 Early exposure to chronic hypoxia induces short- and long-term regulation of hemoglobin gene expression in European sea bass (Dicentrarchus labrax). J. Exp. Biol. 220: 3119–3126. 10.1242/jeb.160713 28646037

[bib11] Del Rio A. M., Davis B. E., Fangue N. A., and Todgham A. E., 2019 Combined effects of warming and hypoxia on early life stage Chinook salmon physiology and development. Conserv. Physiol. 7 10.1093/conphys/coy078 PMC6387995 30834124

[bib12] Ghalambor C. K., McKay J. K., Carroll S. P., and Reznick D. N., 2007 Adaptive *vs.* non-adaptive phenotypic plasticity and the potential for contemporary adaptation in new environments. Funct. Ecol. 21: 394–407. 10.1111/j.1365-2435.2007.01283.x

[bib13] Grek C. L., Newton D. A., Spyropoulos D. D., and Baatz J. E., 2011 Hypoxia Up-Regulates Expression of Hemoglobin in Alveolar Epithelial Cells. Am. J. Respir. Cell Mol. Biol. 44: 439–447. 10.1165/rcmb.2009-0307OC 20508070 PMC3095918

[bib14] Feil R., and Fraga M. F., 2012 Epigenetics and the environment: emerging patterns and implications. Nat. Rev. Genet. 13: 97–109. 10.1038/nrg3142 22215131

[bib15] Gavery M. R., Nichols K. M., Berejikian B. A., Tatara C. P., Goetz G. W., 2019 Temporal dynamics of DNA methylation patterns in response to rearing juvenile steelhead (*Oncorhynchus mykiss*) in a hatchery *vs.* simulated stream environment. Genes (Basel) 10: 356 10.3390/genes10050356 PMC6563097 31075961

[bib16] Greig S. M., Sear D. A., and Carling P. A., 2006 A review of factors influencing the availability of dissolved oxygen to incubating salmonid embryos. Hydrol. Processes 21: 323–334. 10.1002/hyp.6188

[bib17] Greig S., Sear D., and Carling P., 2007 A field-based assessment of oxygen supply to incubating Atlantic salmon (*Salmo salar*) embryos. Hydrol. Processes 21: 3087–3100. 10.1002/hyp.6635

[bib18] Hamor T., and Garside E. T., 1976 Developmental rates of embryos of Atlantic salmon, *Salmo salar* L., in response to various levels of temperature, dissolved oxygen, and water exchange. Can. J. Zool. 54: 1912–1917. 10.1139/z76-221 991016

[bib19] Hancock R. L., Dunne K., Walport L. J., Flashman E., and Kawamura A., 2015 Epigenetic regulation by histone demethylases in hypoxia. Epigenomics 7: 791–811. 10.2217/epi.15.24 25832587

[bib20] Hatzimichael E., Dasoula A., Shah R., Syed N., Papoudou-Bai A., 2010 The prolyl-hydroxylase EGLN3 and not EGLN1 is inactivated by methylation in plasma cell neoplasia. Eur. J. Haematol. 84: 47–51. 10.1111/j.1600-0609.2009.01344.x 19737309

[bib21] Hay N., and Sonenberg N., 2004 Upstream and downstream of mTOR. Genes Dev. 18: 1926–1945. 10.1101/gad.1212704 15314020

[bib22] Ho D. H., and Burggren W. W., 2012 Parental hypoxic exposure confers offspring hypoxia resistance in zebrafish (*Danio rerio*). J. Exp. Biol. 215: 4208–4216. 10.1242/jeb.074781 22899535

[bib23] Iversen M., Finstad B., McKinley R. S., Eliassen R. A., Carlsen K. T., 2005 Stress responses in Atlantic salmon (Salmo salar L.) smolts during commercial well boat transports, and effects on survival after transfer to sea. Aquaculture 243: 373–382. 10.1016/j.aquaculture.2004.10.019

[bib41] Ivan M., Kondo K., Yang H., Kim W., Valiando J., 2001 HIFα targeted for VHL-mediated destruction by proline hydroxylation: Implications for O2 sensing. Science 292: 464–468. 10.1126/science.1059817 11292862

[bib24] Jaakkola P., Mole D. R., Tian Y. M., Wilson M. I., Gielbert J., 2001 Targeting of HIF-alpha to the von Hippel-Lindau ubiquitylation complex by O2-regulated prolyl hydroxylation. Science 292: 468–472. 10.1126/science.1059796 11292861

[bib25] Jaenisch R., and Bird A., 2003 Epigenetic regulation of gene expression: How the genome integrates intrinsic and environmental signals. Nat. Genet. 33: 245–254. 10.1038/ng1089 12610534

[bib26] Janaszak-Jasiecka A., Bartoszewska S., Kochan K., Piotrowski A., Kalinowski L., 2016 miR-429 regulates the transition between Hypoxia-Inducible Factor (HIF)1A and HIF3A expression in human endothelial cells. Sci. Rep. 6: 22775 10.1038/srep22775 26954587 PMC4782134

[bib27] Kvamme B. O., Gaden K., Finne-Fridell F., Niklasson L., and Sundh H., 2013 Modulation of innate immune responses in Atlantic salmon by chronic hypoxia-induces stress. Fish Shellfish Immunol. 34: 55–65. 10.1016/j.fsi.2012.10.006 23085636

[bib28] Langmead B., Trapnell C., Pop M., and Salzberg S. L., 2009 Ultrafast and memory-efficient alignment of short DNA sequences to the human genome. Genome Biol. 10: R25 10.1186/gb-2009-10-3-r25 19261174 PMC2690996

[bib29] Le Luyer J., Laporte M., Beacham T. D., Kaukinen K. H., Withler R. E., 2017 Parallel epigenetic modifications induced by hatchery rearing in a Pacific salmon. Proc. Natl. Acad. Sci. USA 114: 12964–12969. 10.1073/pnas.1711229114 29162695 PMC5724268

[bib30] Li H., Handsaker B., Wysoker A., Fennell T., Ruan J., 2009 The sequence alignment/map format and SAMtools. Bioinformatics 25: 2078–2079. 10.1093/bioinformatics/btp352 19505943 PMC2723002

[bib31] Lien S., Koop B. F., Sandve S. R., Miller J. R., Kent M. P., 2016 The Atlantic salmon genome provides insights into rediploidization. Nature 533: 200–205. 10.1038/nature17164 27088604 PMC8127823

[bib32] Liu J., Dias K., Plagnes-Juan E., Veron V., Panserat S., 2017 a Long-term programming effect of embryonic hypoxia exposure and high-carbohydrate diet at first feeding on glucose metabolism in juvenile rainbow trout. J. Exp. Biol. 220: 3686–3694. 10.1242/jeb.161406 28798080

[bib33] Liu J., Plagnes-Juan E., Geurden I., Panserat S., and Marandel L., 2017 b Exposure to an acute hypoxic stimulus during early life affects the expression of glucose metabolism-related genes at first-feeding in trout. Sci. Rep. 7: 363 10.1038/s41598-017-00458-4 28337034 PMC5428409

[bib34] Love M. I., Huber W., and Anders S., 2014 Moderated estimation of fold change and dispersion for RNA-seq data with DESeq2. Genome Biol. 15: 550 10.1186/s13059-014-0550-8 25516281 PMC4302049

[bib35] Luo W., and Wang Y., 2018 Epigenetic regulators: multifunctional proteins modulating hypoxia-inducible factor-α protein stability and activity. Cell. Mol. Life Sci. 75: 1043–1056. 10.1007/s00018-017-2684-9 29032501 PMC5984203

[bib36] Manchenkov T., Pasillas M. P., Haddad G. G., and Iman F. B., 2015 Novel genes critical for hypoxic preconditioning in zebrafish are regulators of insulin and glucose metabolism. G3 (Bethesda) 5: 1107–1116. 10.1534/g3.115.018010 25840431 PMC4478541

[bib37] Maruyama K., Yasumasu S., and Iuchi I., 2002 Characterization and expression of embryonic and adult globins of the teleost Oryzias latipes (Medaka). J. Biochem. 132: 581–589. 10.1093/oxfordjournals.jbchem.a003260 12359073

[bib38] Maxwell P. H., Wiesener M. S., Chang G. W., Clifford S. C., Vaux E. C., 1999 The tumour suppressor protein VHL targets hypoxia-inducible factors for oxygen-dependent proteolysis. Nature 399: 271–275. 10.1038/20459 10353251

[bib39] Mendelsohn B. A., and Gitlin J. D., 2008 Coordination of development and metabolism in the pre-midblastula transition zebrafish embryo. Dev. Dyn. 237: 1789–1798. 10.1002/dvdy.21584 18521947

[bib42] Moghadam H. K., Johnsen H., Robinson N. A., Anderson Ø., and Jørgensen E. H., 2017 Impacts of early life stress on the methylome and transcriptome of Atlantic salmon. Sci. Rep. 7: 5023 10.1038/s41598-017-05222-2 28694447 PMC5504078

[bib43] Nanduri J., Semenza G. L., and Prabhakar N. R., 2017 Epigenetic changes by DNA methylation in chronic and intermittent hypoxia. Am. J. Physiol. Lung Cell. Mol. Physiol. 313: L1096–L1100. 10.1152/ajplung.00325.2017 28839104 PMC5814703

[bib44] Pan Y. K., Ern R., Morrison P. R., Brauner C. J., and Esbaugh A. J., 2017 Acclimation to prolonged hypoxia alters hemoglobin isoform expression and increases hemoglobin oxygen affinity and aerobic performance in a marine fish. Sci. Rep. 7: 7834 10.1038/s41598-017-07696-6 28798467 PMC5552867

[bib45] Pedersen C. L., 1987 Energy budgets for juvenile rainbow trout at various oxygen concentrations. Aquaculture 62: 289–298. 10.1016/0044-8486(87)90171-2

[bib46] Pfaffl M. W., Tichopad A., Prgomet C., and Neuvians T. P., 2004 Determination of stable housekeeping genes, differentially regulated target genes and sample integrity: BestKeeper – Excel-based tool using pair-wise correlations. Biotechnol. Lett. 26: 509–515. 10.1023/B:BILE.0000019559.84305.47 15127793

[bib47] R Core Team, (2019). R: A Language and Environment for Statistical Computing. Retrieved from https://www.R-project.org/

[bib48] Randall D. J., Perry S. F., and Heming T. A., 1982 Gas transfer and acid/base regulation in salmonids. Comp. Biochem. Physiol. B Biochem. Mol. Biol. 73: 93–103. 10.1016/0305-0491(82)90203-6

[bib49] Remen M., Oppedal F., Torgersen T., Imsland A. K., and Olsen R. E., 2012 Effects of cyclic environmental hypoxia on physiology and feed intake of post-smolt Atlantic salmon: initial responses and acclimation. Aquaculture 326: 148–155. 10.1016/j.aquaculture.2011.11.036

[bib50] Remen M., Aas T. S., Vagseth T., Torgersen T., Olsen R. E., 2014 Production performance of Atlantic salmon (*Salmo salar* L.) postsmols in cyclic hypoxia, and following compensatory growth. Aquacult. Res. 45: 1355–1366. 10.1111/are.12082

[bib51] Richards J. G., 2009 Metabolic and molecular responses of fish to hypoxia, pp. 443–485 in Fish Physiology: Hypoxia, edited by Jeffrey G. R., Anthony P. F., and Colin J. B. Elsevier, Amsterdam, the Netherlands 10.1016/S1546-5098(08)00010-1

[bib52] Robertson C. E., Wright P. A., Koblitz L., and Bernier N. J., 2014 Hypoxia-inducible factor-1 mediates adaptive developmental plasticity of hypoxia tolerance in zebrafish, *Danio rerio* Proceedings of the Royal Society B-Biological Sciences 281 (1786). 10.1098/rspb.2014.0637 PMC4046416 24850928

[bib53] Rombough P. J., 1988 Respiratory gas exchange, aerobic metabolism, and effects of hypoxia during early life. Fish Physiol. 11: 59–161. 10.1016/S1546-5098(08)60199-5

[bib54] Rubin J. F., and Glimsäter C., 1996 Egg-to-fry survival of the sea trout in some streams of Gotland. J. Fish Biol. 48: 585–606. 10.1111/j.1095-8649.1996.tb01454.x

[bib55] Rutjes H. A., Nieveen M. C., Weber R. E., Witte F., and Thillart G. E. E. J. M. V., 2007 Multiple strategies of Lake Victoria cichlids to cope with lifelong hypoxia include hemoglobin switching. Am. J. Physiol. Regul. Integr. Comp. Physiol. 293: R1376–R1383. 10.1152/ajpregu.00536.2006 17626121

[bib56] Samy J. K. A., Mulugeta T. D., Nome T., Sandve S. R., Grammes F., 2017 SalmoBase: an integrated molecular data resource for *Salmonid* species. BMC Genomics 18: 482 10.1186/s12864-017-3877-1 28651544 PMC5485693

[bib57] Shah R., Hatzimichael E., Syed N., Bourantas K. L., and Crook T., 2007 Epigenetic profiling identifies EGLN3 as a frequent target for transcriptional silencing in plasma cell neoplasias. Blood 110: 2132 10.1182/blood.V110.11.2132.213217526861

[bib58] Smith T. G., Robbins P. A., and Ratcliffe P. J., 2008 The human side of hypoxia-inducible factor. Br. J. Haematol. 141: 325–334. 10.1111/j.1365-2141.2008.07029.x 18410568 PMC2408651

[bib59] Solstorm D., Oldham T., Solstorm F., Klebert P., and Stien L. H., 2018 Dissolved oxygen variability in a commercial sea-cage exposes farmed Atlantic salmon to growth limiting conditions. Aquaculture 486: 122–129. 10.1016/j.aquaculture.2017.12.008

[bib60] Stehfest K. M., Carter C. G., McAllister J. D., Ross J. D., and Semmens J. M., 2017 Response of Atlantic salmon Salmo salar to temperature and dissolved oxygen extremes established using animal-borne environmental sensors. Sci. Rep. 7: 4545 10.1038/s41598-017-04806-2 28674437 PMC5495760

[bib61] Szyf M., 2013 DNA Methylation, Behaviour and Early Life Adversity. J. Genet. Genomics 40: 331–338. 10.1016/j.jgg.2013.06.004 23876773

[bib62] The Gene Ontology Consortium, 2019 The Gene Ontology Resource: 20 years and still GOing strong. Nucleic Acids Res. 47: D330–D338. 10.1093/nar/gky1055 30395331 PMC6323945

[bib63] Trapnell C., Pachter L., and Salzberg S. L., 2009 TopHat Discovering splice junctions with RNA-Seq. Bioinformatics 25: 1105–1111. 10.1093/bioinformatics/btp120 19289445 PMC2672628

[bib64] Uren Webster T. M., Rodriguez-Barreto D., Martin S. A. M., Van Oosterhout C., Orozco-terWengel P., 2018 Contrasting effects of acute and chronic stress on transcriptome, epigenome, and immune response of Atlantic salmon. Epigenetics 13: 1191–1207. 10.1080/15592294.2018.1554520 30526303 PMC6986783

[bib65] Vanderplancke G., Claireaux G., Quazuguel P., Madec L., Ferra S., 2015 Hypoxic episode during the larval period has long-term effects on European sea bass juveniles (*Dicentrarchus labrax*). Mar. Biol. 162: 367–376. 10.1007/s00227-014-2601-9

[bib66] Vandesompele J., De Preter K., Pattyn F., Poppe B., Van Roy N., , 2002 Accurate normalization of real-time quantitative RT-PCR data by geometric averaging of multiple internal control genes. Genome Biol. 3: 0034.1. 10.1186/gb-2002-3-7-research0034 PMC126239 12184808

[bib67] Vikeså V., Nankervis L., and Hevrøy E. M., 2017 Appetite, metabolism and growth regulation in Atlantic salmon (Salmo salar L.) exposed to hypoxia at elevated seawater temperature. Aquacult. Res. 48: 4086–4101. 10.1111/are.13229

[bib68] Wang S. Y., Lau K., Lai K., Zhang J., Tse A. C., 2016 Hypoxia causes transgenerational impairments in reproduction of fish. Nat. Commun. 7: 1–9. 10.1038/ncomms12114 PMC4932196 27373813

[bib69] Wang T., Lefevre S., Huong D. T. T., Cong N. V., and Bayley M., 2009 The effects of hypoxia on growth and digestion. Fish Physiol. 27: 361–396. 10.1016/S1546-5098(08)00008-3

[bib70] Wood A. T., Clark T. D., Elliott N. G., Frappell P. B., and Andrewartha S. J., 2019 Physiological effects of dissolved oxygen are stage-specific in incubating Atlantic salmon (*Salmo salar*). J. Comp. Physiol. 189: 109–120. 10.1007/s00360-018-1199-5 30603847

[bib71] Youngson A. F., Malcolm I. A., Thorley J. L., Bacon P. J., and Soulsby C., 2004 Long-residence groundwater effects on incubating salmonid eggs: low hyporheic oxygen impairs embryo development. Can. J. Fish. Aquat. Sci. 61: 2278–2287. 10.1139/f04-217

